# Very-High-Cycle Fatigue Behavior of Inconel 718 Alloy Fabricated by Selective Laser Melting at Elevated Temperature

**DOI:** 10.3390/ma14041001

**Published:** 2021-02-20

**Authors:** Zongxian Song, Wenbin Gao, Dongpo Wang, Zhisheng Wu, Meifang Yan, Liye Huang, Xueli Zhang

**Affiliations:** 1 School of Materials Science and Engineering, Taiyuan University of Science and Technology, Taiyuan 030024, China; songzongxian@tsguas.edu.cn; 2 School of Aeronautics and Astronautics, Tianjin Sino-German University of Applied Sciences, Tianjin 300350, China; sy2430276891@163.com (M.Y.); huangly2021@163.com (L.H.); zxl982511@163.com (X.Z.); 3 School of Materials Science and Engineering, Jiangsu University of Science and Technology, Zhenjiang 212003, China; gaowb@just.edu.cn; 4 School of Materials Science and Engineering, Tianjin University, Tianjin 300350, China; wangdp@tju.edu.cn

**Keywords:** selective laser melting, nickel-based alloy, very-high-cycle fatigue, elevated temperature

## Abstract

This study investigates the very-high-cycle fatigue (VHCF) behavior at elevated temperature (650 °C) of the Inconel 718 alloy fabricated by selective laser melting (SLM). The results are compared with those of the wrought alloy. Large columnar grain with a cellular structure in the grain interior and Laves/δ phases precipitated along the grain boundaries were exhibited in the SLM alloy, while fine equiaxed grains were present in the wrought alloy. The elevated temperature had a minor effect on the fatigue resistance in the regime below 10^8^ cycles for the SLM alloy but significantly reduced the fatigue strength in the VHCF regime above 10^8^ cycles. Both the SLM and wrought specimens exhibited similar fatigue resistance in the fatigue life regime of fewer than 10^7^–10^8^ cycles at elevated temperature, and the surface initiation mechanism was dominant in both alloys. In a VHCF regime above 10^7^–10^8^ cycles at elevated temperature, the wrought material exhibited slightly better fatigue resistance than the SLM alloy. All fatigue cracks are initiated from the internal defects or the microstructure discontinuities. The precipitation of Laves and δ phases is examined after fatigue tests at high temperatures, and the effect of microstructure on the formation and the propagation of the microstructural small cracks is also discussed.

## 1. Introduction

Inconel 718 (IN718) alloy is one of the most widely used nickel-iron-based superalloys in the high temperature (up to 650 °C) structure applications due to its high strength, as well as the high creep, oxidation and corrosion resistance over a wide temperature range [1]. The main strengthening phases are the coherent γ″ (Ni_3_Nb) and γ′ (Ni_3_Al) precipitates in the γ matrix. The brittle Laves phase (Ni,Fe,Cr)_2_(Nb,Mo,Ti) and δ phase (Ni_3_Nb), usually formed along the grain boundaries, are detrimental to the mechanical properties [2]. The traditional manufacturing technology of nickel-based superalloy parts includes forging, casting and powder metallurgy. However, the poor machinability of IN718 due to the high strength makes it costly in the application of complex shapes or cavities, such as turbine blade engine fuel nozzle [3]. Selective laser melting (SLM) is one of the most attractive additive manufacturing (AM) techniques due to its high forming precision, high density and excellent mechanical properties [4].

Numerous studies focus on the randomly dispersed defects, the microstructure heterogeneity and the anisotropic mechanical properties for SLM materials [5,6]. Although IN718 is widely used in the high-temperature environment and is often subjected to cyclic loading, previous studies mainly focused on the fatigue behavior at room temperature (RT). During the SLM process, due to the interaction between powder and heat source, a large number of defects are formed, such as pores, unfused defects and unmelted powder [7,8]. Defects usually act as the source of fatigue cracks and reduce the fatigue life. Johnson et al. found that the fatigue property of the AM specimen was lower than that of the forged specimen due to the presence of the carbide, oxide inclusion and porosity defects near the surface [9]. Pei et al. also reported that SLM IN718 had inferior fatigue properties than the forged counterpart, and the difference was more obvious with the increase in cycles, indicating more sensitivity to defects for SLM alloy in the very-high-cycle fatigue (VHCF) regime [10]. However, Gribbin found that the role of porosity present in the direct metal laser sintered IN718 specimens is not as significant at low strain amplitudes as it is at high strain amplitudes [11].

Limited studies exist regarding the effect of microstructure and defects on the fatigue behavior at elevated temperature, especially in the very-high-cycle fatigue (VHCF) regime. Nezhadfar et al. investigated the low cycle fatigue resistance of SLM IN718 at RT and 650 °C and found that SLM specimens possessed slightly lower fatigue resistance at RT but exhibited a comparable fatigue strength at elevated temperature [12]. They ascribed the similar fatigue behavior at 650 °C to the same initiation sites of surface oxide films formed during the fatigue testing, which could retard the growth of the microstructurally short cracks. Zhao et al. investigated the initiation and early-stage crack growth under VHCF at RT, 750 °C and 850 °C on directionally solidified Ni-base superalloy [13]. They found that the fatigue strength at 850 °C was higher than that at 750 °C and RT in the VHCF regime due to the higher threshold for propagating the early-stage crack at 850 °C. Muhammad et al. found that the fatigue cracks of SLM IN718 after VHCF tests at RT, initiated from intragranular slip bands near or at the surface, and strong anisotropy in the fatigue resistance was exhibited in the SLM alloy due to the presence of columnar grains along the build direction [14].

In this paper, the VHCF behavior is investigated at room and elevated (650 °C) temperatures and is compared to that of the traditional wrought material. Analysis techniques used a variety of microstructure characterization, tensile tests and ultrasonic fatigue tests. The fatigue behavior is discussed with regard to the microstructure evolution during the fatigue testing at elevated temperature. The results of this work provide a better understanding of the VHCF behavior of SLM IN718 alloy used in high-temperature applications.

## 2. Materials and Methods

Additively manufactured nickel-based superalloy IN718 was fabricated by selective laser melting (SLM) of IN718 powders (Beijing Institute of Aeronautical Materials, Beijing, China) using an EOS M290 machine (EOS, Munich, Germany) equipped with a 400 W fiber laser. The particles with a size of 15–45 μm were obtained by atomization method, and the chemical composition of IN718 powder is listed in Table 1. The printing process was conducted under argon shielding gas to protect the molten pool. The SLM parameters were as follows: laser power of 260 W, scanning speed of 1000 mm/s, hatch spacing of 0.11 mm and layer thickness of 40 μm. The cross-direction raster scanning (CDRS) strategy was adopted to fabricate several cylindrical rods with a diameter of 10 mm and a height of 80 mm. This scanning strategy contained a first contouring and the following printing in the core of the specimen [12]. The scanning direction rotated 90° for each subsequent layer. The as-deposited IN718 was subjected to the AMS 5663 heat treatment specification of the first solid solution annealing at 980 °C for 1 h and the second double aging at 720 °C for 8 h and then at 620 °C for 8 h [15]. Wrought IN718 alloys were manufactured by vacuum induction melting and vacuum arc remelting (VIM + VAR) processes. The materials were hot forged into round bars at a temperature of 1040–1065 °C for the first forging and at a temperature of 1010–1040 °C for the second forging and then subjected to the same heat treatment specification with the SLM alloys.

Metallographic samples were ground with a series of SiC papers (Jiangsu East Grinding Co., Ltd., JiangDu, China) and then polished down to 1 μm diamond suspension. Chemical etching was conducted in an aqueous solution of 1.5 g CuSO_4_ + 20 mL HCl + 10 mL ethanol (Changsheng Chemical Co. Ltd., JinZhou, China) for 10 s. The microstructures were examined by an optical microscopy (OM) and a JSM-7800F field emission scanning electron microscope (SEM) (JEOL, Tokyo, Japan) equipped with energy-disperse X-ray spectroscopy (EDS) (JEOL, Tokyo, Japan). Electron backscatter diffraction (EBSD) examinations for the SLM IN718 and wrought IN718 were also performed with a step size of 1.5 μm. The surfaces selected for EBSD examinations were perpendicular to the build direction or the forging direction. EBSD specimens were prepared by electropolishing in a mixed solution of HClO_4_ (Tianjin Damao Chemical Reagent Factory, Tianjin, China) and ethanol (10:90 vol%) under a voltage of 30 V for 20 s.

**Figure 1:**
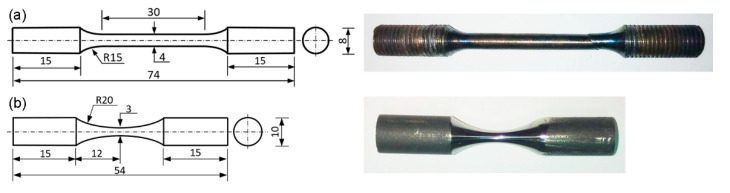
Geometric characteristics of (**a**) tensile and (**b**) ultrasonic fatigue samples (all dimensions in mm).

Tensile tests were conducted based on the GB/T 228.2-2015 standard [16] using an INSTRON 5982 machine (Instron Corporation, Canton, MA, USA). The strain rate was preset as 3.3 × 10^−5^·s^−1^ before yielding and 3.3 × 10^−4^·s^−1^ after yielding. The loading axis was parallel to either the building direction or the forging direction. The geometry and size of the tensile sample are shown in Figure 1a. The cylindrical tensile samples were polished with a series of SiC papers to remove any impurity or discontinuity. The samples tested at 650 °C were heat-preserved at 650 °C for 10 min before loading. Three tests were conducted for each condition, and a total of 12 samples were strained to failure.

USF-300 ultrasonic fatigue testing system (Tianjin Yipu Technology Co., Ltd., Tianjin, China), composed of an ultrasonic generator, a piezoelectric ceramics tranducer and a vibrating hone, was used to perform the very-high-cycle fatigue (VHCF) tests at a frequency of 20 kHz and at a stress ratio of −1. In order to obtain a larger stress amplification factor, the specimen was designed as a dog-bone shape with variable cross-section. The geometry and size were exhibited in Figure 1b. The sample was designed based on the resonance requirements, and the maximum stress was located in the middle section with zero displacement. The sample was subjected to the symmetrical tension-compression fatigue loads (i.e., R = −1). Detailed calculation equations can be found in Zhao’s previous publication [13]. A mirror-like surface was obtained by a series of SiC papers before testing. The loading direction was parallel to the build direction for the SLM specimens and to the forging direction for the wrought specimens. The stress amplitude, σ_a_, ranged from 410 MPa to 570 MPa to establish the S-N curves. The specimens tested at 650 °C were heated by an induction coil. The tests would be terminated automatically when the fatigue cracks caused a resonance frequency change of 5%, or when the fatigue life reached 1 × 10^9^ cycles. A total of 43 fatigue samples were fabricated to obtain the VHCF S-N curves. The fracture surfaces were observed by a JSM-7800F SEM, and the compositions of the local regions near the crack initiation sites were determined by EDS.

## 3. Results

### 3.1. Microstructure Characterization

**Figure 2:**
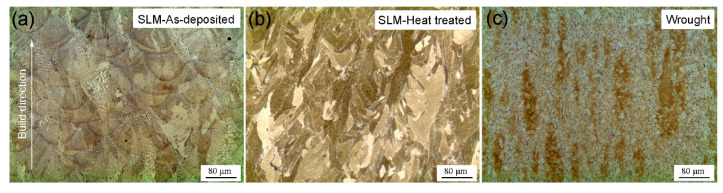
Optical microscopy (OM) images for the planes parallel to the build direction for (**a**) as-deposited selective laser melting (SLM) IN718, (**b**) SLM plus heat treatment (SLM-HT) and (**c**) wrought specimens.

Figure 2 shows the optical micrographs of as-deposited, SLM plus heat treatment (SLM-HT) and wrought counterpart specimens. Layered structures, as a result of the laser beam’s alternating motion along the plane perpendicular to the build direction, are present in the SLM specimen (Figure 2a). The average layer thickness was about 40 μm. Columnar grains orientated in the build direction are observed in the form of fine dendrites. After heat treatment, the columnar dendritic arrays still existed (Figure 2b). Recrystallization did not occur due to the low peak temperature in the present study (980 °C), which is lower than the reported recrystallization temperature (1020 °C) for the wrought IN718 [17]. Small elliptical grains, along with large amounts of rod precipitates, are present in the wrought IN718 specimen (Figure 2c).

**Figure 3:**
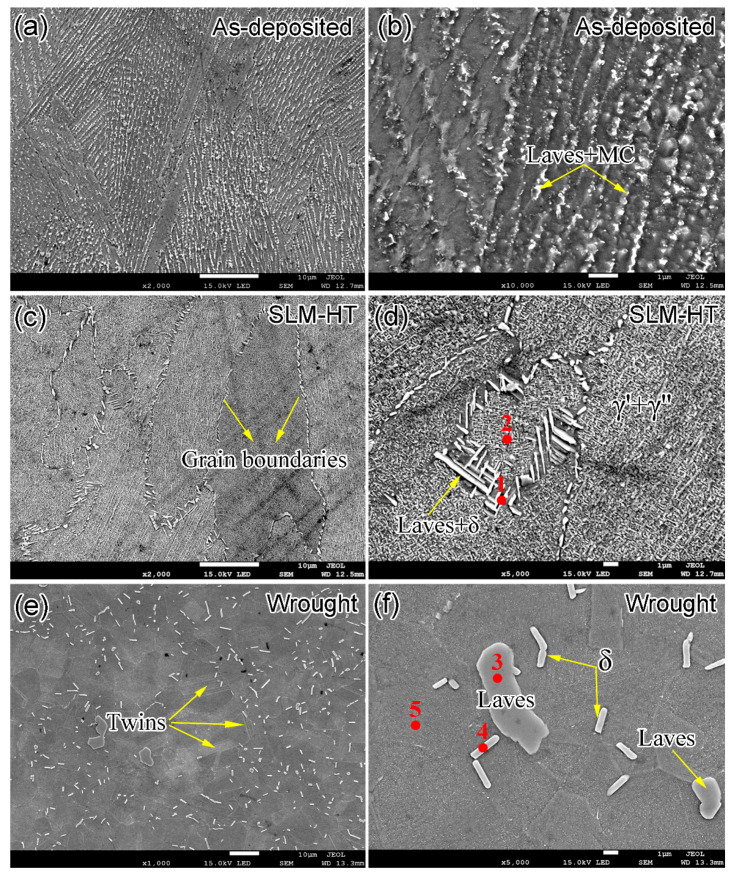
SEM images showing the microstructure of (**a**,**b**) as-deposited SLM IN718, (**c**,**d**) SLM plus heat treatment (SLM-HT) and (**e**,**f**) the wrought counterpart. Solid red circles are EDS test positions and the results are listed in Table 2.

Figure 3 shows the SEM images for the as-deposited, SLM-HT and wrought IN718 specimens. The microstructure of as-deposited specimen was composed of γ matrix, and large amounts of long-striped Laves phase and MC particles (e.g., NbC) in the interdendritic regions (Figure 3a,b). The grain boundaries are not clearly visible, but a number of columnar/cellular sub-grains are present in the interior of grains. The typical width of the sub-granular structure with Nb-rich boundaries ranges from 500 nm to 1 μm (Figure 3b). The Nb-rich boundaries are the remnants of the as-deposited dendritic microstructure [18]. No δ, γ′ or γ″ precipitate was found in the as-deposited specimen, and the absence of these precipitates is related to the high cooling rate in the SLM process, which was up to 10^3^–10^5^ K/s. The short time of phase transformation did not allow the nucleation and growth for these precipitates. Figure 3c,d shows the microstructure of SLM alloy after heat treatment. Grain boundaries, decorated by large amounts of needle-like Laves and δ precipitates, are clearly observed. Compared to the as-deposited alloy, the number of Laves phase decreased after heat treatment, and Nb diffused from the dissolution of Laves phases to the interdendritic areas, resulting in the formation of δ precipitates. The microstructure mainly consisted of Laves and δ precipitates along the grain boundaries, and γ′/γ″ precipitates above the γ matrix in the interior of the grains. The short acicular δ phase, as determined by EDS analysis shown in Table 2, possessed a length of less than 5 μm, and was found close to the Laves phase due to the Nb diffusion from Laves precipitates during the aging process at 980 °C. The presence of δ precipitates along grain boundaries was also in accordance with the fact that the fastest precipitation of the δ phase occurred at about 900 °C [19]. The microstructure of wrought IN718 mainly consisted of large blocky and rod precipitates both along the grain boundaries and in the interior of the grains (Figure 3e,f). The chemical compositions of these two major types of precipitates were further determined by EDS analysis, as listed in Table 2. Results show that the large blocky precipitate with 43.5% Ni and 22.9% Nb is Laves phase, and the rod precipitates with 63.5% Ni and 20.6% Nb is δ phase. The statistical chemical compositions are consistent with previous research about SLM IN718 [20]. The rod δ precipitates possessed a width of about 0.5 μm and a length up to 10 μm. Moreover, some twins are observed. The presence of twins is due to the low stacking-fault energy at high temperatures (i.e., 1000 °C) during the hot forging process. Therefore, equiaxed grains were formed for the wrought IN718, and this is confirmed by the EBSD inverse polar figure (IPF) shown below.

**Figure 4:**
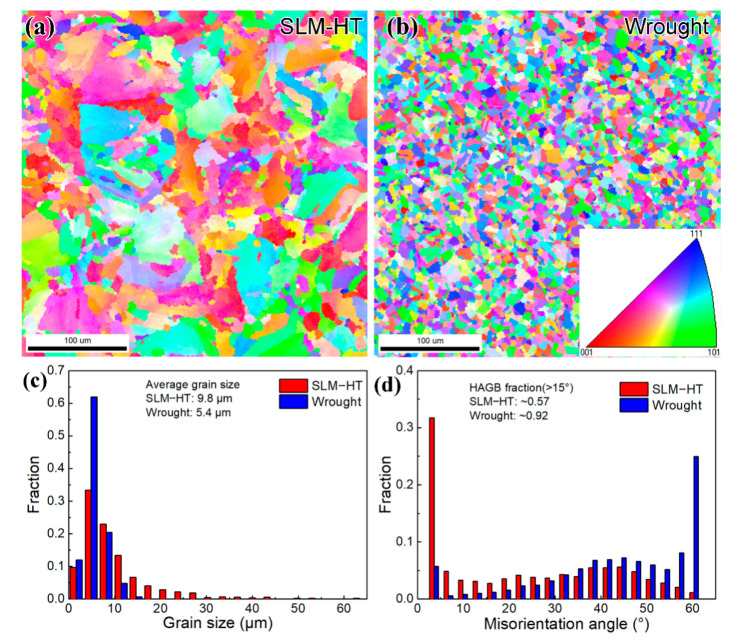
EBSD inverse polar figure maps of (**a**) SLM-HT and (**b**) wrought specimens, (**c**) grain size distribution and (**d**) grain boundary misorientation distribution. The scanning planes were perpendicular to the build direction for the SLM-HT specimen and the forging direction for the wrought counterpart.

Figure 4a,b show the EBSD inverse polar figure (IPF) maps for heat-treated SLM IN718 (SLM-HT) and wrought counterpart, respectively. It should be noted that the scanning planes for both specimens are perpendicular to the build direction or forging direction. Therefore, the common columnar grain structure for SLM IN718 is not exhibited in the present study. In general, SLM IN718 exhibited larger and more nonuniform grains than those of the wrought counterpart. Fine recrystallized grains with a diameter of about 5 μm were present in the wrought alloy. It has been reported that the static recrystallization temperature of wrought IN718 alloy is about 1020 °C [17]. As the peak temperature during the hot forging was 1065 °C, large amounts of recrystallized grains were exhibited in the present study. The grain size for the SLM specimen ranged from 2 to 60 μm, while the maximum grain size for the wrought specimen was only 15 μm (Figure 4c). The statistical average grain size was 9.8 μm for SLM IN718 alloy and 5.4 μm for the wrought specimen. Figure 4d shows the grain boundary misorientation angle results for the two specimens. SLM IN718 possessed the most low-angle grain boundaries (LAGB) with misorientation angles less than 15°, whereas the wrought specimen had more high-angle grain boundaries (HAGB). The fraction of HAGB was 0.57 for the SLM IN718 specimen and 0.92 for the wrought specimen, respectively.

### 3.2. Tensile Behavior

**Figure 5:**
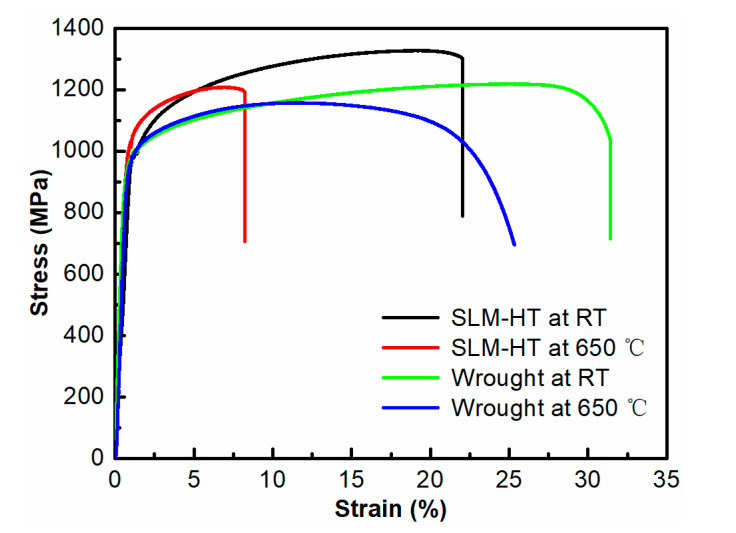
Tensile stress-strain curves of SLM-HT and wrought IN718 specimens tested at RT and 650 °C. The tensile curves of SLM alloy at RT and wrought material at 650 °C are cited from the author’s previous publications [23,24]. Adapted with permission from ref. [23]. Copyright 2020 Nanjing University of Aeronautics and Astronautics. Adapted with permission from ref. [24]. Copyright 2020 Shanghai Research Institute of Materials.

Figure 5 shows the stress–strain curves for SLM plus heat treatment (SLM-HT) and wrought specimens tested at RT and 650 °C. Yield strength (YS), ultimate tensile strength (UTS) and elongation are listed in Table 3. The SLM-HT specimens exhibited slightly higher UTS but lower elongations than those of wrought specimens tested in both RT and 650 °C environments. In particular, the UTS was 1298 MPa at RT and 1196 MPa at 650 °C for SLM-HT alloy, whereas it decreased to 1195 MPa and 1165 MPa for the wrought alloy. The high strength of the SLM alloy is ascribed to the microstructural refinement of the fine columnar/cellular structure. The strength and ductility decreased at elevated temperatures for both the SLM and wrought specimens. This can be explained by the thermally aided movement of dislocation and the weakening bond strength due to the increase in the mean free path of atoms at elevated temperature [21]. The elongation was 17.0% at RT and 6.9% at 650 °C for the SLM-HT specimens, and it increased to 30.3% and 22.4% for the wrought specimens. The high strength and low plasticity exhibited in the SLM-HT alloy is related to the high supersaturation of solute atoms, the large amounts of fine celluar subgrains within the columnar grains and the defects (e.g., lack of fusion defects and porosity) during the SLM process [7]. In addition, the wrought sample exhibited larger plastic deformation after yielding than the SLM-HT sample at 650 °C. This indicates that the effect of temperature on plasticity was more pronounced for the SLM-HT specimens than that for the wrought specimens. Compared with the tensile results tested at RT, there was a large decrease in the elongation for the SLM alloy when tested at elevated temperatures. This is consistent with the work examining the creep properties between SLM and wrought IN 718 alloys at 700 °C. That work shows that the SLM IN718 exhibited poor creep resistance and plasticity due to the high residual stress, large columnar structure, and continuous coarse Laves or δ phases at grain boundaries [22].

### 3.3. Fatigue Behavior

**Figure 6:**
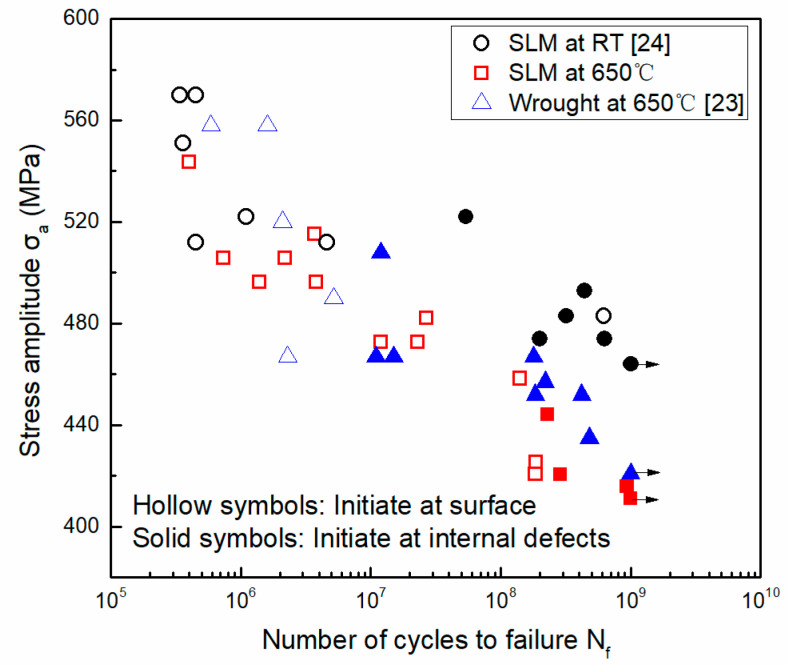
S-N curves of SLM and wrought specimens tested at RT and 650 °C. The S-N curves of SLM alloy at RT and wrought material at 650 °C are cited from the author’s previous publications [23,24]. Adapted with permission from ref. [23]. Copyright 2020 Nanjing University of Aeronautics and Astronautics., Adapted with permission from ref. [24]. Copyright 2020 Shanghai Research Institute of Materials.

Figure 6 shows the fatigue test results for SLM specimens tested at RT and 650 °C and wrought specimens tested at 650 °C. The arrows indicate that the specimens did not fail after 1 × 10^9^ cycles. With the decrease in the loading stress amplitude, all three S-N curves exhibit a continuous downward trend, and there was no traditional engineering fatigue limit after 10^7^ cycles. The specimens still suffered fatigue fracture after 10^7^ or even 10^9^ cycles. The data appear to have little scattering in the fatigue life below 10^8^ cycles, but exhibit a large difference in the fatigue life regime above 10^8^ cycles. Specifically, the fatigue strength was the highest for the SLM specimens tested at RT, followed by the wrought specimens tested at 650 °C and then the SLM specimens tested at 650 °C. Comparing the two S-N curves of the SLM specimens tested at RT and 650 °C reveals that the S-N curves show little difference below 10^7^ cycles, but exhibit a large difference when the fatigue life is above 10^8^ cycles. This indicates that temperature has a minor effect on the fatigue resistance in the short fatigue life regime, but exhibits a large effect in the long fatigue life regime for the SLM material. Furthermore, the temperature effect is more pronounced with the increase in the fatigue life above 10^8^ cycles.

Comparing the two curves of the wrought alloy and the SLM alloy tested at high temperature (i.e., 650 °C) reveals that there was little difference of the fatigue strength below 10^8^ cycles, while the wrought specimens possessed a slightly better fatigue resistance than the SLM specimens in the region above 10^8^ cycles. The small difference in the S-N curves below 10^8^ cycles between the SLM and the wrought specimens is partly due to the same heat treatment specifications used in both of the two materials, which can lead to similar types of second phases in both materials.

### 3.4. Fractography

**Figure 7:**
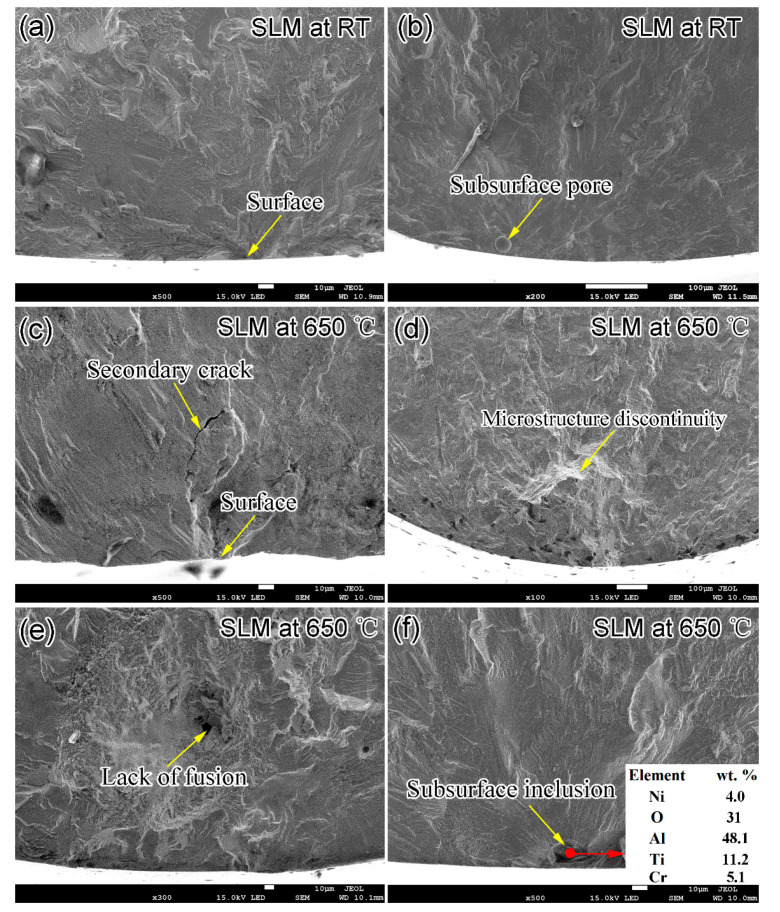
Representative SEM fractographic images of SLM IN718 specimens tested at (**a**,**b**) RT and (**c**,**d**) 650 °C: (**a**) σ_a_ = 570 MPa, N_f_ = 4.4 × 10^5^; (**b**) σ_a_ = 480 MPa, N_f_ = 3.1 × 10^8^; (**c**) σ_a_ = 472 MPa, N_f_ = 2.3 × 10^7^; (**d**) σ_a_ = 420 MPa, N_f_ = 2.9 × 10^8^; (**e**) σ_a_ = 444 MPa, N_f_ = 2.3 × 10^8^; (**f**) σa = 416 MPa, Nf = 9.4 × 10^8^.

**Figure 8:**
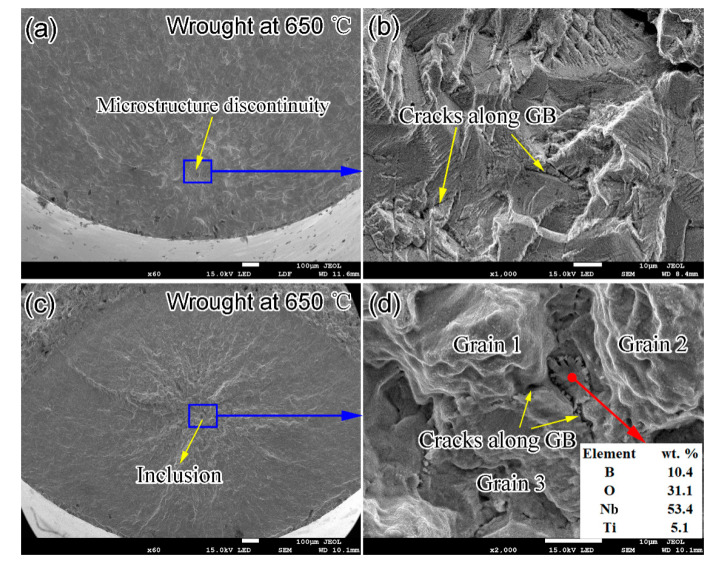
Representative SEM fractographic images of wrought IN718 specimens tested at 650 °C: (**a**,**b**) σ_a_ = 457MPa, N_f_ = 2.2 × 10^8^; (**c**,**d**) σa = 440 MPa, Nf = 4.5 × 10^8^.

Figure 7 and Figure 8 are representative SEM fractography images of the SLM and wrought IN718 specimens, respectively. Two main types of fatigue crack sources, namely surface for the short fatigue life specimens and subsurface pore for the long fatigue life specimens, were detected for the SLM specimens tested at RT (Figure 7a,b). One typical subsurface sphere pore with a diameter of 28 μm is shown in Figure 7b. The presence of pores in SLM specimens is attributed to the entrapped gas during SLM process, which resulted from either the gas in the initial powders or the inert atmosphere (e.g., argon). The pore-induced fatigue crack initiation mechanism prevailed in the SLM specimens, especially in the specimens with fatigue lives above 10^8^ cycles. Figure 7c–f show the representative fatigue crack initiation sites for the SLM specimens tested at 650 °C. Similarly to the SLM specimens tested at RT, surface defects dominate the fatigue behaviors in the fatigue life regime below 10^8^ cycles. However, three main types of fatigue crack sources, namely microstructure discontinuity (Figure 7d), lack of fusion (Figure 7e) and subsurface inclusion (Figure 7f), are distinguished in the specimens with a fatigue life of more than 10^8^ cycles. The microstructure discontinuity is believed to be either the brittle second phase such as Laves phase and δ phase or the multiple crystallographic facets. The presence of crystallographic facets are the results of the initiation and growth of the microstructurally small cracks and are strong evidence for the fatigue crack initiation at persistent slip bands (PSB) [14]. Figure 7e shows a fatigue crack initiation site of a cavity with an irregular shape due to the lack of fusion. The cavity with sharp corners seen in the lack of fusion defect is the most detrimental internal defect for the fatigue resistance. Figure 7f exhibits a crack initiation site of a subsurface inclusion, which is rich in O, Al and Ti examined by EDS. Since there are few internal inclusion defects due to the stable fabrication process and proper parameters during SLM, the inclusion-induced fatigue crack initiation mechanism is only found in one specimen. Interestingly, no fatigue crack is found to initiate at internal pores for the SLM specimens tested at 650 °C, while the pore-induced crack initiation mechanism prevailed in the SLM specimens tested at RT (Figure 7b). This might be related to the lesser degree of the stress concentration of pores with spherical or elliptical morphology at high temperature. Gas pores are less detrimental than other internal defects. It is the internal defects, such as lack of fusion defects and microstructure discontinuities, that dominate the fatigue behavior at high temperature.

Figure 8 shows the representative SEM fractography images of wrought specimens tested at 650 °C. The fatigue cracks originated from the surface defects for the specimens in the fatigue life below 10^7^ cycles. However, the fatigue crack initiation was triggered by microstructure discontinuities in the fatigue life regime above 10^7^ cycles. The identification of the crack initiation sites for the wrought material with dense and fine grains is difficult. Similar to the internal initiation sites in the SLM specimens tested at 650 °C, the fatigue cracks for the wrought specimens initiate at the microstructure discontinuities, including the facets (Figure 8b) or the second phases/inclusions decorated along the grain boundaries (Figure 8d). Large amounts of smooth and flat facets with striations are observed in the vicinity of the point of crack initiation (Figure 8b). Figure 8d shows a fatigue crack source of an inclusion at the triple point of three grains, and the EDS analysis indicates that it is rich in B, O and Nb. A number of secondary cracks are present on the fracture surfaces, and the crack growth mode is intergranular. This feature is ascribed to the fine grains with grain boundaries decorated by large amounts of brittle Laves and δ phases (Figure 3f), which can break under cyclic deformation at high temperature and lead to the intergranular secondary cracks.

## 4. Discussion

### 4.1. Temperature Effect on the High Cycle Fatigue Behavior for SLM IN718 Alloy

There is little difference between the two S-N curves for the SLM material tested at room and elevated temperatures in the fatigue life regime below 10^8^ cycles, indicating that the elevated temperature has little effect. However, a large difference in fatigue resistance is exhibited in the S-N curves in the long fatigue life regime above 10^8^ cycles. The effect of elevated temperature on fatigue lives can be described as the loss of strength and the formation of oxidizing film on the surface of the specimens. The material softening has a negative effect on the fatigue resistance, while the oxidizing film on the surface can improve the fatigue resistance and promote the transition of the fatigue crack initiation site from the surface to the internal defects. Kawagoishi et al. [25] investigated the effect of oxide film formed at 600 °C on the fatigue property of IN718 forgings after 10^7^ cycles. Slip lines and microcracks, indicative of the initial stage of the fatigue crack growth, were observed below the stripped oxide films. They concluded that the oxide films could cover the initial crack and played a positive role in inhibiting the surface crack initiation and improving the fatigue property. It is noteworthy to point out that the aforementioned analysis is based on the assumption that there is no defect in the interior. In fact, a number of internal defects are present in the SLM alloy. Thus, the positive effect of the oxide film is not considered as an important factor in the case of the internal-defect-induced initiation. The present study suggests the similar fatigue S-N curves of SLM specimens with fatigue lives less than 10^8^ cycles tested at room and elevated temperatures. This phenomenon can be explained by the short testing time at elevated temperature due to the large testing frequency of 20 kHz. Specifically, the testing time even for 10^8^ cycles was only 80 min, and this short time is not sufficient to form thick oxide film to inhibit the initial microcracks. For high cycle and very-high-cycle fatigue, the life consumed at the crack initiation stage accounts for most of the total fatigue life, even more than 95% of the total fatigue life [26]. As such, the elevated temperature has little effect on the high cycle fatigue resistance of the specimens with fatigue cracks initiating at the surface.

In fatigue life regimes above 10^8^ cycles, both the SLM specimens tested at room and elevated temperatures had an internal defect-induced initiation mechanism, and a higher fatigue strength is exhibited in the specimens tested at RT than those tested at elevated temperatures. The inferior fatigue resistance at elevated temperature is related to the material softening and the microstructure deterioration. Laves and δ phases possess a wide-ranging melting and precipitation temperature from 650 to 1100 °C [20]. Consequently, more Laves and δ phases can precipitate during the fatigue testing, especially in the VHCF regime. The SLM material tested at elevated temperature has lower strength and appears to deform more easily, leading to the accumulation of large amounts of the persistent slip bands around the internal defects or the brittle second phases (e.g., Laves and δ phases) at grain boundaries. Then, the localized regions of high dislocation density caused by the stress concentration sites can promote the formation of cavities, which evolve into the initial small fatigue crack.

Furthermore, the fatigue crack was initiated at the subsurface pore for the specimens tested at RT, but at the microstructure discontinuities (e.g., internal lack of fusion or inclusion defects) at elevated temperature (Figure 7). The subsurface-pore-induced initiation mechanism was not found in any SLM specimens at elevated temperature. This phenomenon suggests that the role of the microstructure discontinuities and internal lack of fusion defects outweighs the pore defects in high-temperature fatigue testing. There exists a competition mechanism of the fatigue crack initiation in the different internal defects. The inclusion defects with an irregular shape deteriorate the fatigue resistance most severely due to the large local stress concentration and the thermal stress resulting from the inconsistent deformation between the inclusion and the matrix. A sphere pore has less stress concentration than the inclusion and does not act as a fatigue initiation source at elevated temperature. Because of the rare inclusion in the present SLM alloy due to the proper fabrication parameters, the microstructure discontinuities are common crack initiation sites in the VHCF regime at elevated temperature due to the accumulation of the incoherent cyclic strain in these regions.

### 4.2. Comparison of the Fatigue Resistance between SLM and Wrought IN718 Alloys at Elevated Temperature

Both SLM and wrought specimens exhibit similar fatigue resistance in the fatigue life regime of less than 10^8^ cycles at elevated temperature, and surface initiation mechanism is dominant in both alloys. This finding is consistent with the comparison of the low cycle fatigue behavior (less than 10^6^ cycles) of the wrought and the IN718 fabricated by laser beam directed energy deposition (LB-DED) at 650 °C [12]. The authors of that study found that the cracks of both the wrought and LB-DED specimens initiated from the surface, and the defects in LB-DED alloy did not affect the fatigue behavior.

In the VHCF regime above 10^8^ cycles at elevated temperature, the wrought material exhibited slightly better fatigue resistance than the SLM alloy. Based on the fracture surface examinations, an interesting finding is that for the SLM specimens with fatigue life below 10^8^ cycles and the wrought specimens with fatigue life below 10^7^ cycles, all the fatigue cracks initiated from the surface. In the regime above 10^7^–10^8^ cycles, however, all fatigue cracks were initiated from the internal defects (e.g., lack of fusion defects and inclusion) and the microstructure discontinuities (e.g., Laves phase and δ phase, or crystallographic facets), regardless of the fabrication processes and the test temperatures, indicating the defect and microstructure dominant failure in the VHCF regime. The transition of the two fatigue crack initiation modes was located at about 10^8^ cycles for the SLM alloy and at 10^7^ cycles for the wrought alloy. The same conclusion can also be reached in the study of the high cycle fatigue for the SLM and wrought IN718 materials [27]. The main type of internal defect in the present study is entrapped pore, which plays a less critical role in VHCF than the inclusion defect. In the absence of voids and inclusions for the wrought alloy, microstructure discontinuities (smooth and flat facets shown in Figure 8) dominate the crack initiation behavior. The crystallographic facets were also observed in the very high cycle fatigue of a nickel-based single-crystal superalloy tested at 1000 °C and were found in the localized regions of high dislocation density induced by the slip of γ/γ’ microstructure [28]. In sum, the few internal inclusion and lack of fusion defects in the present SLM material can explain its slightly lower fatigue resistance than the wrought alloy.

**Figure 9:**
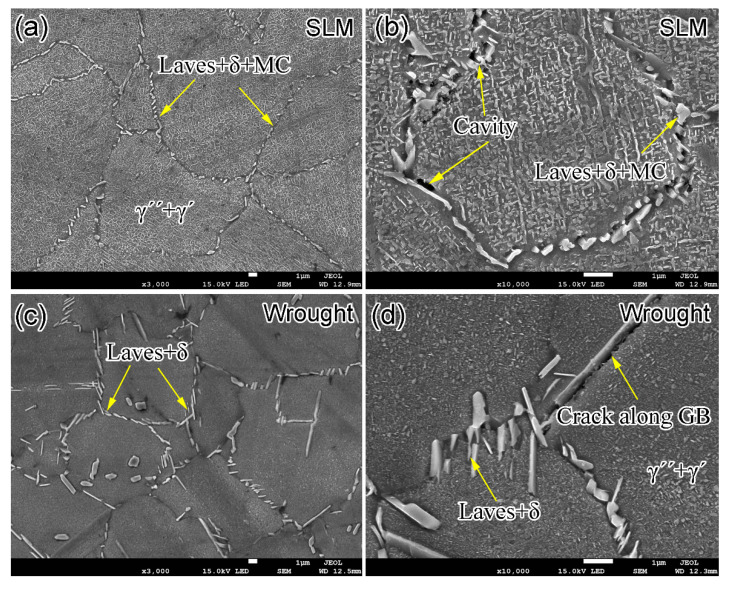
SEM images showing microstructure and microcracks of (**a**,**b**) SLM IN718 run-out specimen (σ_a_ = 411 MPa, N_f_ = 1 × 10^9^) and (**c**,**d**) the wrought run-out specimen (σ_a_ = 421 MPa, N_f_ = 1 × 10^9^) after fatigue tests at 650 °C. The examined surfaces are located in the middle of the samples and are perpendicular to the loading direction.

In order to investigate the microstructure evolution and the fatigue crack initiation mechanism, SEM examinations are conducted on the planes cut from the run-out (10^9^ cycles) SLM and wrought specimens (Figure 9). As mentioned above, Laves and δ phases can precipitate and grow during the high-temperature fatigue testing process. No obvious differences can be found between the microstructures before (Figure 3c) and after (Figure 9a) fatigue test for the SLM specimen. However, as shown in Figure 9c, more rod or needle-like Laves and δ phases can be observed in the wrought specimen after experiencing a long fatigue life of 10^9^ cycle (the corresponding during time is 14 h) than those in the same material before the fatigue test (Figure 3e). The majority of these precipitates are δ phase. Since the Nb-rich Laves and δ phases are prone to nucleate along the grain boundaries due to the lower activation energy barrier at elevated temperature, grain refinement can lead to the increase in the length and area of the grain boundaries, thereby promoting the Laves and δ phases. Recrystallized grains with a small grain size of about 5 μm were formed in the wrought alloy during the hot forging process (Figure 4c). The grain size for the SLM material, however, was is about 10 μm in the plane perpendicular to the build direction, and it was larger in the plane with coarse columnar grains parallel to the build direction. As such, the wrought alloy provides far more grain boundaries acting as the nucleation sites of the Laves and δ phases. This can explain the more and larger Laves and δ phases in the wrought alloy after fatigue testing. The loss of coherent reinforcement between the brittle Laves or δ phases and the matrix under cyclic stress at elevated temperature can lead to grain boundary embrittlement and provide the crack propagation path [29]. This is confirmed by the SEM examinations for the run-out specimens. Figure 9b shows the cavities in the interface of the Laves or δ phases and the matrix along the grain boundaries. The linkage of these cavities is critical for the formation of the microstructural small fatigue crack. A microcrack along the grain boundary is also observed in the wrought specimen (Figure 9d). Based on the analysis above, the size and distribution of Laves or δ phases play an important role in the fatigue crack initiation in the VHCF regime at elevated temperatures.

For the crystallographic cracking observed in the long fatigue life regime above 10^7^–10^8^ cycles, the grain boundary and the adjacent grain orientations can significantly affect the propagation of the microstructural small fatigue crack. In situ observation of the fatigue short crack propagation behavior of SLM IN718 alloy shows that the blocking effect of grain boundaries decelerated the fatigue crack growth rate remarkably [30]. Previous studies show that the twin boundary and high angle grain boundary are effective obstacles to the short crack growth due to the large crack path defection [31]. As discussed before, the wrought alloy has more grain boundaries due to the small grain size than the SLM alloy. Moreover, most of the grain boundaries possess high misorientation angles for the wrought alloy, while most of the grain boundaries have low misorientation angles less than 15° for the SLM material. A large number of mechanical twin boundaries are observed in the wrought alloy. Therefore, the small grain size and the high fraction of HAGB and twin boundaries contribute to impeding the microstructural small fatigue crack propagation.

In sum, the internal defects and the microstructure discontinuity dominate the fatigue crack initiation behavior of both the SLM and wrought alloys in VHCF regime at elevated temperatures. More Laves and δ phases are precipitated along the grain boundaries due to the grain refinement in the wrought alloy and act as the potential fatigue crack sources. In addition, the impeding effect of HAGB and twin boundaries in the wrought alloy decelerate the propagation rate of the microcracks. All these factors lead to slightly better VHCF resistance in the wrought alloy than that in the SLM material at elevated temperature.

It should be noted that the SLM material is subjected to the same heat treatment specification (solution at 980 °C for 1 h and double aging at 720 °C for 8 h and then at 620 °C for 8 h, STA) traditionally used for the wrought materials, and this heat treatment is not always the best one for the SLM alloy. Previous work by Yu et al. has shown that post-heat-treatment specifications have a great effect on the microstructure evolution and the mechanical properties for the Inconel 718 alloy fabricated with laser-directed energy deposition [17]. They found that the additive manufactured IN718 alloy subjected to the homogenization at 1100 °C for 1.5 h prior to the traditional solution plus double aging (HSTA) possessed the best tensile and fracture resistance. Specifically, fracture toughness K_IC_ of STA and HSTA samples increased by 55.8% and 90.8% compared with the as-fabricated sample. Therefore, we believe that the large difference in the microstructure and mechanical properties among the alloys subjected to various post heat treatments will significantly affect the VHCF behavior of the SLM material. However, the exact correlation is beyond the scope of the present study. The authors intend to build this correlation in future efforts.

## 5. Conclusions

Very-high-cycle fatigue (VHCF) behavior of SLM IN718 is investigated at elevated temperature (650 °C) and is compared to the wrought counterparts. The microstructure evolution and fatigue failure mechanism are also characterized. Major findings are as follows:

1. The SLM material exhibits slightly higher strength but lower elongations than those of wrought specimens tested in both room and elevated temperatures. The high strength and low plasticity exhibited in the SLM alloy is related to the high supersaturation of solute atoms, the large amounts of fine cellular subgrains within the columnar grains and the defects (e.g., lack of fusion defects and porosity) during the SLM process.
2. Compared with the fatigue behavior for the SLM alloy at RT, the elevated temperature has little effect on the fatigue resistance in the regime below 10^8^ cycles, but significantly reduces the fatigue strength in the VHCF regime above 10^8^ cycles.
3. Both SLM and wrought specimens exhibit similar fatigue resistance in the fatigue life regime of fewer than 10^8^ cycles at elevated temperature, and the surface initiation mechanism is dominant in both alloys. In VHCF regime above 10^8^ cycles at elevated temperature, the wrought material exhibits slightly better fatigue resistance than the SLM alloy. All fatigue cracks are initiated from the internal defects (e.g., lack of fusion defects and inclusion) and the microstructure discontinuities (e.g., Laves phase and δ phase, or crystallographic facets), regardless of the fabrication processes, indicating the microstructure-dominant failure in VHCF regime.
4. The internal defects and the microstructure discontinuity dominate the fatigue crack initiation behavior of both the SLM and wrought alloys in the VHCF regime at elevated temperatures. The wrought alloy exhibits a slightly better VHCF resistance at elevated temperature than the SLM material, due to the combined effects of the more potential fatigue crack sources (Laves and δ phases precipitated along the grain boundaries during the fatigue testing) and the more HAGB and twin boundaries impeding the propagation of the microcracks in the wrought material.

## Figures and Tables

**Table 1:** Chemical composition of IN718 alloy powder and the wrought alloy (wt.%).

Materials	Cr	Ni	Nb	Mo	Ti	Al	C	Si	Cu	Fe
IN718 alloy powder	18.52	52.44	5.25	3.03	0.98	0.52	0.04	<0.1	<0.1	Balance
Wrought alloy	19.00	53.00	5.30	3.00	1.00	0.50	0.05	-	-	Balance

**Table 2:** EDS results (wt.) of positions as indicated in Figure 3.

Position	Ni	Nb	Cr	Fe	Mo	Ti	Al
1	26.2	43.9	12.0	10.9	-	6.8	-
2	54.2	5.3	18.4	17.0	3.6	0.9	0.7
3	43.5	22.9	16.7	9.1	4.8	2.6	0.4
4	63.5	20.6	5.1	5.8	-	2.4	-
5	54.2	4.6	18.4	17.7	3.4	1.1	0.6

**Table 3:** Summary of tensile results for SLM-HT and wrought IN718 specimens tested at RT and 650 °C.

Condition	Test Temperature	YS (MPa)	UTS (MPa)	Elongation (%)
SLM-HT	RT	992 ± 2	1298 ± 29	17.0 ± 3.2
	650 °C	983 ± 28	1196 ± 12	6.9 ± 0.7
Wrought	RT	934 ± 9	1195 ± 25	30.3 ± 1.1
	650 °C	936 ± 6	1165 ± 6	22.4 ± 2.3

## Data Availability

Data available on request due to restrictions eg privacy or ethical. The data presented in this study are available on request from the corresponding author. The data are not publicly available due to [Management requirements of the grant fund].

## References

[1] Firoz R., Basantia S., Khutia N., Bar H., Sivaprasad S., Murthy G. (2020). Effect of microstructural constituents on mechanical properties and fracture toughness of Inconel 718 with anomalous deformation behavior at 650 °C. J. Alloys Compd..

[2] Ardi D.T. (2020). Effects of post-processing route on fatigue performance of laser powder bed fusion Inconel 718. Addit. Manuf..

[3] Trosch T., Strößner J. (2016). Microstructure and mechanical properties of selective laser melted Inconel 718 compared to forging and casting. Mater. Lett..

[4] Wan H., Zhou Z., Li C., Chen G., Zhang G. (2019). Effect of scanning strategy on mechanical properties of selective laser melted Inconel. Mater. Sci. Eng. A.

[5] Witkin D.B., Patel D. (2020). Influence of surface conditions and specimen orientation on high cycle fatigue properties of Inconel 718 pre-pared by laser powder bed fusion. Int. J. Fatigue.

[6] Watring D.S., Benzing J.T., Hrabe N., Spear A.D. (2020). Effects of laser-energy density and build orientation on the structure–property relationships in as-built Inconel 718 manufactured by laser powder bed fusion. Addit. Manuf..

[7] Sanaei N., Fatemi A. (2020). Defects in additive manufactured metals and their effect on fatigue performance: A state-of-the-art review. Prog. Mater. Sci..

[8] Hosseini E., Popovich V. (2019). A review of mechanical properties of additively manufactured Inconel. Addit. Manuf..

[9] Johnson A.S., Shao S., Shamsaei N., Thompson S.M., Bian L. (2016). Microstructure, Fatigue Behavior, and Failure Mechanisms of Direct Laser-Deposited Inconel. JOM.

[10] Pei C., Shi D. (2019). Assessment of mechanical properties and fatigue performance of a selective laser melted nickel-base superalloy In-conel 718. Mater. Sci. Eng. A Struct..

[11] Gribbin S., Bicknell J., Jorgensen L., Tsukrov I., Knezevic M. (2016). Low cycle fatigue behavior of direct metal laser sintered Inconel alloy. Int. J. Fatigue.

[12] Nezhadfar P., Johnson A.S., Shamsaei N. (2020). Fatigue behavior and microstructural evolution of additively manufactured Inconel 718 under cyclic loading at elevated temperature. Int. J. Fatigue.

[13] Zhao Z., Zhang F., Dong C., Yang X., Chen B. (2020). Initiation and Early-Stage Growth of Internal Fatigue Cracking under Very-High-Cycle Fatigue Regime at High Temperature. Met. Mater. Trans. A.

[14] Muhammad M., Frye P., Simsiriwong J., Shao S., Shamsaei N. (2021). An investigation into the effects of cyclic strain rate on the high cycle and very high cycle fatigue behaviors of wrought and additively manufactured Inconel. Int. J. Fatigue.

[15] (2009). Nickel Alloy, Corrosion and Heat-Resistant, Bars, Forgings and Rings 52 Ni-19 Cr-3.0 Mo-5.1 Cb (Nb)-0.9 Ti-0.50 Al-18 Fe Con-Sumable Electrode or Vacuum Induction Melted 1775 °F (968 °C) Solution and Precipitation Heat Treated, SAE AMS 5663 M 2009, SAE international Group, Warrendale, USA. http://www.sae.org/technical/standards/AMS5663M.

[16] (2015). Metallic Materials-Tensile Testing-Part 2: Method of Test at Elevated Temperature, GB/T 228.2 2015, Standardization Administration of China: Beijing, China. http://std.samr.gov.cn/gb/search/gbDetailed?id=71F772D80910D3A7E05397BE0A0AB82A.

[17] Yu X., Lin X., Liu F., Wang L., Tang Y., Li J., Zhang S., Huang W. (2020). Influence of post-heat-treatment on the microstructure and fracture toughness properties of Inconel 718 fabricated with laser directed energy deposition additive manufacturing. Mater. Sci. Eng. A.

[18] Zhao L., Tan Y., Shi S., You X., Li P., Cui C. (2020). Microsegregation behavior of Inconel 718 superalloy prepared by electron beam smelting layered solidification technology. J. Alloys Compd..

[19] Tucho W.M., Cuvillier P., Sjolyst-Kverneland A., Hansen V. (2017). Microstructure and hardness studies of Inconel 718 manufactured by selective laser melting before and after solution heat treatment. Mater. Sci. Eng. A.

[20] Yu X., Lin X., Tan H., Hu Y., Zhang S., Liu F., Yang H., Huang W. (2020). Microstructure and fatigue crack growth behavior of Inconel 718 superalloy manufactured by laser directed energy deposition. Int. J. Fatigue.

[21] Wan H.Y., Luo Y.W., Zhang B., Song Z.M., Wang L.Y., Zhou Z.J., Li C.P., Chen G.F., Zhang G.P. (2020). Effects of surface roughness and build thickness on fatigue properties of selective laser melted Inconel 718 at 650 °C. Int. J. Fatigue.

[22] Xu Z., Cao L., Zhu Q., Guo C., Li X., Hu X., Yu Z. (2020). Creep property of Inconel 718 superalloy produced by selective laser melting compared to forging. Mater. Sci. Eng. A.

[23] Song Z.X., Qi H. (2020). Study on Ultrahigh Cycle Fatigue Performance of GH4169 Nickel-based Alloy at 650 °C. Trans. Nanjing Univ. Aeronaut. Astronaut..

[24] Song Z.X., Wang D. (2020). Ultrahigh cycle fatigue performance of GH4169 alloy by selective laser melting. Mater. Mech. Eng..

[25] Kawagoishi N., Chen Q., Nisitani H. (2000). Fatigue strength of Inconel 718 at elevated temperatures. Fatigue Fract. Eng. Mater. Struct..

[26] Zhu M.-L., Jin L., Xuan F.-Z. (2018). Fatigue life and mechanistic modeling of interior micro-defect induced cracking in high cycle and very high cycle regimes. Acta Mater..

[27] Yang K., Huang Q., Wang Q., Chen Q. (2020). Competing crack initiation behaviors of a laser additively manufactured nickel-based superalloy in high and very high cycle fatigue regimes. Int. J. Fatigue.

[28] Cervellon A., Hémery S., Kürnsteiner P., Gault B., Kontis P., Cormier J. (2020). Crack initiation mechanisms during very high cycle fatigue of Ni-based single crystal superalloys at high temperature. Acta Mater..

[29] Sui S., Chen J., Fan E., Yang H., Lin X., Huang W. (2017). The influence of Laves phases on the high-cycle fatigue behavior of laser additive manufactured Inconel. Mater. Sci. Eng. A.

[30] Ma X.F., Zhai H.L. (2020). Fatigue short crack propagation behavior of selective laser melted Inconel 718 alloy by in-situ SEM study: In-fluence of orientation and temperature. Int. J. Fatigue.

[31] Liu S., Li H., Qin C., Zong R., Fang X. (2020). The effect of energy density on texture and mechanical anisotropy in selective laser melted Inconel. Mater. Des..

